# Medical errors, medical negligence and defensive medicine: A narrative review

**DOI:** 10.1016/j.clinsp.2022.100053

**Published:** 2022-05-28

**Authors:** Ivan Dieb Miziara, Carmen Silvia Molleis Galego Miziara

**Affiliations:** a Department of Legal Medicine, Ethics and Occupational Health, Faculdade de Medicina da Universidade de São Paulo, São Paulo, SP, Brazil; b Discipline of Legal Medicine and Bioethics, Faculdade de Medicina do ABC, Santo André, SP, Brazil

**Keywords:** Medical errors, Medical liability, Negligence, Defensive medicine

## Abstract

Error in medicine and medical liability has a long history dating back to Antiquity. During the 19th Century, most lawsuits related to errors in treating surgical problems were settled. However, in the first half of the 20th Century, lawsuits claimed that mistakes were related to the doctor's action: the doctor made something wrong (errors of commission). In Brazil, medical error is defined as inappropriate conduct, including negligence and recklessness, that causes harm to the patient. The physician's fear of being suited is the reason for some practice named defensive Medicine (D.M.), defined as ordering unnecessary tests and procedures or avoiding treatments for patients considered at high-risk. Thus, this narrative review aims to analyze and describe the relationship between medical errors, medical negligence, and the practice of D.M. So, the authors propose procedures and attitudes to avoid medical errors and the approach of D.M.: a national focus to create leadership and research tools to enhance the knowledge base about patient safety; a reporting system that would help to identify and learn from errors; the use of a computer-based protocol reminder; some technological devices to help the medical practice (electronic prescribing and information technology systems); creating risk management programs in hospitals. Therefore, the authors conclude that the most critical attitude to avoid medical liability is a good and ethical medical practice with the proper use of technology, based on knowledge of scientific evidence and ethical principles of medicine - for the benefit of patients.

## HIGHLIGHTS

• This article defines medical errors and medical responsibility from a Brazilian perspective.
• This article calls attention to the risks of medical liability and the unethical use of defensive medicine.
• The authors propose some procedures and attitudes to avoid medical errors like the use of technology at the bedside and computer-based protocols.
• The authors state that a good and ethical medical practice can avoid medical liability.

## Introduction

Error in medicine and questions about medical liability has a long history dating back to Antiquity. In Mesopotamia, there was already the Code of Hammurabi (1792‒1750 BC). This code dealt with the mistakes made by doctors in the exercise of their profession. One of the first reports of errors in medicine was made by British legal scholar Sir William Blackstone. In 1765, he published a compendium of legal principles entitled Commentaries on the Laws of England. He refers to “Mala Praxis”, which he defines as “neglect or unskillful management of a physician or surgeon”. The modern word “malpractice” is derived^1^ from this term. Since then, in 1794, “the first recorded medical malpractice lawsuit in the U.S. takes place in Connecticut where a patient died of a surgical complication”.^2^ De Ville^3^ stated, “in 1871 the Medical and Surgical Report recounted a daring solution to a malpractice charge”. One patient told his surgeon that he would sue him due to a poorly healed fracture. The surgeon offered to correct the defect and resolve the problem. The patient refused, so the surgeon immobilized him and successfully operated. The patient then decided to drop all charges. According to De Ville^3^ “it might be characterized as an early case of defensive medicine”.

The problem evolved during the 19^th^ Century through lawyers who acted aggressively, with most lawsuits related to errors in treating fractures, dislocations, and amputations.^1^

Lawsuits for alleged medical errors continued during the first half of the 20^th^ Century. Most of them claimed that mistakes were related to the doctor's action: the doctor made something wrong, so-called errors of commission, “such as causing injuries due to too much radiation (during radiographs examination), complications of surgical treatment, and diagnostic failures”.^1^^,^^4^ From the 1950s onwards, there was a transition from errors of action to errors of omission. The doctor failed to do what was correct for that particular case. For example, from the early 1970s to the late 1980s, the number of lawsuits in the U.S. alleging failure to diagnose cancer increased by 50%.^3^ Berlin1 stated that “a 1991 study disclosed that 75% of all adverse events due to negligence committed in New York hospitals in the late 1980s involved diagnostic mishaps, usually the result of a physician's failure to do something”. In the meantime, “malpractice claims dramatically increased between the 1960s and 1980s reaching 15 claims out of 100 physicians in a given year, with a doubling in payouts”.^5^

At this point, someone can argue: what really is a medical error?

Some authors define a medical error as “the failure of a planned action to be completed as intended or the use of a wrong plan to achieve an aim”.^6^ In Brazil, medical error is defined as inappropriate conduct due to negligence, recklessness, or malpractice that causes harm to the patient. It is a sign of the evolution of the “error concept”, which encompasses all aspects of behavior capable of causing damage to the patient.

In summary, allegations of negligence against physicians suffered a transformation over the last decades: in the beginning, patients sued them for doing something wrong; now, the doctors began being sued for failing to do something right.

As a result, contingency plans “often arise within a clinician when faced with thoughts of shame, or embarrassment”^1^^,^^5^ following an accusation of wrongdoing by patients or family, medical errors, and other lapses in judgment. Also, there has been a tendency to react with overdiagnosis, and unnecessary surgeries, which may be harmful to patients.^1^^,^^5^ This approach to extreme care is psychologically soothing. But it also can be profit-motivated. And worst: medical students and residents can observe this practice (e.g., so-called defensive medicine) in their training, perpetuating the problem.

Thus, this narrative review aims to analyze and describe the relationship between medical errors, medical negligence, and defensive medicine and propose some procedures and attitudes to avoid the mistakes and the practice of defensive medicine.

## Methods

An extensive search query was utilized, analyzing results from journal articles regarding medical error, medical negligence, and defensive medicine. A MEDLINE, PubMed, and Cochrane Library literature search were conducted with search terms “defensive medicine” AND “medical malpractice” OR “medical errors”. All articles were analyzed for this narrative review, but the authors chose 34 of them that we believed were essential to explaining the authors’ point of view.

## Discussion

Physicians are accustomed to operating within multi-level guidelines, but errors can arise in planning actions or executing them. According to Oyebode,^6^ errors include “improper transfusions, surgical injuries, wrong-site surgeries, suicides, restraint-related injuries or death, falls, burns, pressure ulcers, and mistaken patient identities”. The most affected settings where these errors occur are intensive care units, operating rooms, and emergency departments.^6^ Medication error is the most common and preventable cause of patient injury, and “the rates of medication errors in pediatric settings appear to be up to three times the rates in adult settings, mainly due to parental drug administration”.^6^

It is important to note that “there are two models of human error causation, namely the person approach and the systems approach”.^6^ The person approach calls attention to individual action, including forgetfulness, inattention, or moral failure. On the other hand, the system approach regards the work's conditions as the source of errors, aiming to understand “the origin of errors and building defenses to prevent them or mitigate their effects”.^6^ It is crucial to know that both systems are complementary and that human rather than technical failures are the sources of most medical errors. They represent a severe threat to potentially hazardous systems.^7^

Oyebode^6^ remarks that the “most common systems failure identified as underlying clinical errors are failures in the dissemination of drug knowledge and inadequate availability of patient information such as test results necessary for safe treatment”. Other causes reported are the disregard of guidelines and policies, unavailability of equipment, “production pressure and hectic schedules”.^8^ Fatigue is a non-negligible factor that needs to be taken into account. Shift work of 24h or more “was more likely to be associated with medical errors than shorter shifts”.^6^^,^^9^

## The rise of defensive medicine

In this scenario, some pathways are intended to prevent error by negligence, primarily by surgeons, in the 21^st^ Century. First, they began to use a type of conduct called “Defensive Medicine” (D.M.). As stated by Hellinger and Encinosa,^10^ “the most damaging attribute of our medical malpractice system is not that it fails to compensate victims or to deter poor performance but that it promotes the practice of “defensive medicine”. Borgan et al.^11^ defined defensive medicine as “the routine medical care to avoid or reduce the risk of real or perceived future legal consequences”. Frati P et al.^12^ stated that “D.M. has been defined as ordering tests, procedures, and visits or avoiding treatments for patients considered at high-risk, to prevent malpractice claims”.

The American Medical Association (AMA) has defined defensive medicine as the “performance of diagnostic tests and treatments which, but for the treat of a malpractice action would not have been done”.^3^

In the last decades, the culture of practice of D.M. spread worldwide due to an increasing number of lawsuits against physicians in all medical specialties. As a result, the physicians used D.M. “to lessen their exposure to medical malpractice litigation” or “by fear of malpractice litigation”.^12^^,^^13^

The specialties most affected by claims are in the U.S and Brazil. were Plastic Surgery, General Surgery, Gynecology, and Dermatology.^14^^,^^15^ In Brazil, in a study carried out in Santa Catarina, in 2005‒2009, the medical specialties with the highest number of litigation in court for medical errors were gynecology, anesthesiology, and general surgery.^14^ In general, in Brazil, “lawsuits against physicians have increased by 1,600%, compared with that in the previous decade, and this is a crucial concern”.^15^

Otherwise, Frati P et al.^12^ pointed out that “several studies have highlighted how lawsuits negatively impact physicians, causing them stress, thereby jeopardizing their future performance”. In addition, it creates a “significant pressure on health professionals, particularly in some specialized branches more exposed to this risk”. De Ville^3^ observes that “physicians are likely to look to the law first, not afterward, and are often preoccupied with maintaining the safest legal procedure possible”. In turn, Frati P et al.^12^ emphasize that “there is no evidence in the literature that a fear of being sued is useful for reducing the medical error rate”.

As far as the authors know, D.M. has two primary forms. An active form, also called “positive”, is when the physician orders extra tests and procedures. The other is “passive or negative” when avoiding high-risk patients and methods. Someone can also categorize defensive practices into two distinct patterns: “assurance practices that unnecessary over-investigate lower-risk patients, and avoidance practices that aim to avoid intervention in the care of higher-risk patients”.^13^ Finally, Baungaart et al.^14^ note that there are other forms different from fear of litigation, which is applied by “self-protective motives”, and they can be grouped in “four categories: fear of patient dissatisfaction; fear of overlooking a severe diagnosis; fear of negative publicity and unconscious defensive medicine”.

In this perspective, exposure to lawsuits has made physicians more careful in their actions and procedures to prevent medical claims, “rather than to promote the patient's best interest”, disregarding medical ethics.^13^ As adequately stated by Hermer & Brody,^17^ “while perhaps not 'unnecessary' care, defensive medicine is meant more to offer economical and psychological benefit to the physician than to the patient”. Again, this disregards medical ethics and the benefit of the patients.

Another problem is that D.M. is not innocuous or harmless. As physicians involved in any surgery may be sued when executing surgical treatment or preoperative activities, the result is the request for unnecessary tests before the procedures with consequent enhancement of the entire process.

## A burden to the health system

In a universal public health system, as in England or Brazil, this means considerable resources, burdening the whole system and harming many other patients who may be left without care. Furthermore, Garattini, Padula & Mannucci^18^ stated in 2020, that “the broad impact of defensive medicine” also includes indirect costs induced by physician's stress, time, and reputation loss. In addition, the authors say that “redundant D.M. practices induced by the threat of medical liability are expected to increase total health care expenditures”. In a developing country like Brazil, with scarce public resources destinated for the health system, it becomes a severe problem affecting all the Community.^16^

The essential principle of ethical behavior in medical practice is to act to the patient's benefit. But it also includes the use of all communities, that is, public health ethics focusing on collective aspects, such as sharing risks and benefits.^13^ Thus, beyond the individual indirect costs, there is a high cost to all the patients that are hampered by the scarcity of public resources.

Kapp^19^ pointed out that D.M. “constitutes bad medical practice, drives up health care costs, depends on medical specialty and a physician's own prior experience as a malpractice defendant”. Although he stated that D.M. is a “rational response to actual legal risks confronting physicians”, he also assumed that D.M. “varies depending on a jurisdiction's particular tort law climate”.^19^

## Medical responsibility

The legal culture in each country towards medical errors and medical responsibility affects how and how D.M. is practiced. In Brazil, for example, it must be clear that medical responsibility is subjective; the doctor should care for the patient but not cure. Moreover, the cure depends on several other individual and collective factors, not just on the doctor's performance. Therefore, it is necessary to characterize the existence of fault on the part of the professional to confirm the presence of an error in medical practice. And guilt, in turn, is manifested by inappropriate medical conduct based on negligence, recklessness, or malpractice. The proof of error is made through the expert examination requested by the judge, who will decide based on this expert analysis.

But, as far as the authors know, in the U.S. and other countries, the medical responsibility is objective; there is a compromise with cure and with a good result – independently of any factor strange to the doctor-patient relationship. Moreover, their legal system “is based on the premise of trial advocacy, which relies on the adversarial arrangement of opposing parties, a judge, and potentially a jury. The jury serves as the decider of fact, whereas the judges decide all questions of law”.^20^

In Brazilian Criminal Law, the doctor can also be called upon in articles 129 (bodily injury) and 121 (to kill someone if the patient dies) of the Penal Code. Whether the doctor has a “non-observance of the professional technical rule” in both cases will be observed. If so, the penalty will be increased. It is essential to point out that “non-observance of professional technical rule” means that the doctor did not follow the protocols and guidelines recommended for each case. Of course, the procedures and protocols are built on the best available scientific evidence.

## Ethical aspects of defensive medicine

Defensive medicine is a medical, legal, and moral problem.^20^, ^21^, ^22^ Someone could contest the power of the state to interfere in medical practice,^21^ furthermore in front of the Criminal Law. But It must be clear that “it is morally permitted for governments to enforce contracts that citizens record as binding among them”.^20^ As Engelhardt^20^ says, “currently, there are no questions that the practice of medicine is controlled through law and regulation”.

However, somebody should view bioethical principles in a relational way.^23^ Respect “principle of beneficence”, for example, must be applied in favor of the individual and regarding the social benefits of all communities.

It is important to note that D.M. in positive (when additional procedures are performed without proven necessity) or negative (when high-risk patients and methods are avoided) form is firmly questioned morally and ethically. Frierson and Joshi^24^ remark that “the duty in medical practice begins when a doctor-patient relationship is established”. In the case of a psychiatrist-patient relationship, for example, when the doctor predicts the possibility of being sued, its improper termination could constitute abandonment. It is unethical behavior and a kind of D.M.

The authors pointed out above that physicians in the U.S. and other countries, in the past decades, “have long believed that they must practice defensive medicine to diminish litigation risk”.^17^ The focus of these physicians is centered on themselves and not on their patients. In other words, they are not acting to benefit the patients, disregarding basic principles like do not cause harm to them or respecting their autonomy.^23^^,^^24^^,^^25^ They do not listen to their patients but convince them that unnecessary exams are “necessary”. It is a kind of fraud. In summary, from this point of view, D.M. is unethical since it disregards actions for the benefit of the patients, adds avoidable risks to patients, and increases costs to society and public health.

Besides, the practice of D.M. does not have the strength to prevent a lawsuit. For example, since defensive medicine became part of medical malpractice 45-years ago, medical errors have increased1; D.M. is not a solution for posterior medical litigation. As stated by Williams et al.,^26^ “This practice (D.M.) does not necessarily prevent malpractice claims and more importantly, neither does it equate to good medical practice, with some leading to poor outcomes”. Unnecessary exams and tests imply overdiagnosis and overtreatment. It is “a new kind of error of Commission”.^1^ Errors of omission (failure to diagnose) declined because of the use of D.M. On the other hand, unfortunately, errors of commission, overdiagnosis, and overtreatment appear on the rise.

Committing an error has devastating consequences for the physician, personally and professionally. To be suspended from practice, convicted, or increased surveillance about his professional performance are some of the worst effects. Colleagues may regard him as incompetent or careless. Hospitals may suspend him to practice. Thus, the fear of being perceived “as a lesser-quality physician” supports the survival of D.M.^1^

## Are there possible solutions to prevent medical errors?

First of all, to answer this question, the authors have to pay attention to other correlated questions, such as individual motivation to perform at the best level of his capacity, professional culture, and social constraints within the workplace. The staff and gestures are really committed to making changes that could prevent errors? If so, are they interested in proscribing procedures of D.M. in their environment? Are they committed to improving health care quality?

Oyebode^6^ states that “there are numerous proposed strategies for reducing the incidence of clinical errors”. The authors can cite a “national focus to create leadership and research tools to enhance the knowledge base about patient safety”. The authors must create a reporting system “that would help identify and learn from errors”,^6^ that would help to ensure that root-cause analysis, hidden during malpractice litigation, will be discovered and prevented. Although reporting systems should be controversial, and "there is little good evidence that errors identification systems are of much use in teaching residents”,^6^ it is evident that a change of culture is more than necessary nowadays. It is also essential to create work teams to improve patient safety, especially in emergency settings.

Since McDonald^27^ proposed the use of computer-based protocol reminders in the mid-1970s, and it proved to be effective in reducing errors, irrespective of the seniority of the clinician, some practical measures can be adopted, such as the use of voice-recognition technology for radiology reports.^28^ In addition, electronic prescribing and information technology systems are methods for reducing prescribing errors that might be effective,^29^ including in intensive care units.^30^

## Improving health care quality

It is essential to note that many studies of malpractice deterrence suggest that a “higher risk of malpractice liability is not significantly associated with improved health care quality”.^31^ In other words, the risk of liability can be itself an adverse event, perpetuating the practices of D.M.

In turn, the authors think that some actions must be taken. As the authors pointed out above, we must act to improve technologies to avoid risks to the patients or build strategies to improve the doctor-patient relationship. It is important to note that “some errors involve momentary or inadvertent lapses at the individual clinician level”.^32^^,^^33^

The authors must invest in creating risk management programs in the study's hospitals. As Kohn et al.^32^ state, “Originating with the increase in liability risk in the mid-1970s, hospital risk management programs have long been associated with reducing institutional liability and financial loss control”. It includes the identification of risks before the events and containing them after. In this perspective, the education of staff and patients is also essential. “Educational efforts tend to focus on reviewing state statutes on informed consent, presentations by the hospital's defense counsel, and programs on medical and legal topics for physicians”.^32^

On the other hand, two problems can be avoided in preventing to use of D.M. First: as Mello et al.^31^ pointed out, “hospitals might be able to implement systems to identify some such errors before they cause harm, but other errors are not amenable to the kind of conscious precaution taking (at either the hospital or the physician level) on which the deterrence model relies”. The other is uncertainty about aspects involving medical responsibility. Many physicians “complain that they do not know what negligence is – i.e., precisely what the law requires in a given situation”.^31^ That is the way the education of the staff is essential. If a physician does not know what negligence is, D.M. seems to him an easy shortcut to avoid it.

Kohn et al.^32^ add that “although effort has been made to move toward ‘primary’ risk management that would focus on preventing adverse events from occurring, risk management is still focused largely on loss control”. Someone could argue that it is a matter of perspective. In other words, where should I put my efforts and my focus?

Kohn et al.^32^ also say that “incident reporting systems are intended to include major events such as surgical mishaps”, and minor incidents have been underreported, mainly because “largely slips, falls, and medication errors that may have little consequence”.^31^ Otherwise, “although risk management committees include a medical staff member, risk management has not been embraced at the organizational leadership level in its broadest sense of patient safety ‒ protecting patients from any accidental injury”. That is, the authors must change the culture within the organizations that control hospitals and other clinics.

In the case of adverse events or errors, Morris et al.^34^ suggest that “we must create a safety culture within a culture of quality”. The authors also build a series of practical points that must be implemented, such as “create a culture that rewards event reporting as a valued task, a culture that holds the reporter blameless for the report, but individuals accountable for the event”. They propose that “we must change our systems and redesign our work” at the institutional level. They reinforce the necessity of moving professionals to the bedside and minimizing nonclinical distractions. In addition, they add: “We must create a workplace that allows the professional to evolve from an individual who records medical information to an individual who processes medical information. We must rapidly integrate new technology that provides bedside electronic data capture and order entry. We must develop the software and systems that predict and prevent adverse events before they occur”.

Another necessary action regarding avoiding medical liability is the performance of an autopsy in the cases of the suspect of medical error. In Brazil, that procedure is made by the Medico-Legal Institute (IML), and they are called “suspicious death”. Kohn et al.^32^ state that “unexpected findings at autopsy are an excellent way to refine clinical judgment and identify misdiagnosis. Lundberg cites a 40 percent discrepancy between antemortem and postmortem diagnoses”. The authors say that “when autopsies are completed, their value in improving care depends on reports reaching clinicians promptly. Yet, many hospitals report long delays (several weeks or more) before clinicians receive autopsy reports. In general, rapid improvement requires shortening the cycle time between investigation and feedback to caregivers and managers. Timeliness in autopsy reporting is representative of all data gathering activities intended for quality improvement and reduction of errors”.

Finally, from the authors’ point of view, the most important solution to prevent medical litigation and the use of D.M. is a good and ethical medical practice with the proper use of technology. A medical practice based on knowledge of scientific evidence and ethical principles of medicine ‒ for the benefit of patients. Nonetheless, “the factors that predict that a patient will resort to litigation include a prior poor relationship with the clinician and the feeling that the patient is not being kept informed”.^6^ On the other hand, the authors must encourage a more open physician-patient relationship, with better communication and respect, with physicians listening to their patients before trying to convince them. It is not a new or innovative proposal but is more than efficient. After all, the places are different, but the patients are the same everywhere.

## Conclusions

Medical errors have several causes, mainly disregarding guidelines and protocols of safety, failures in the dissemination of drug knowledge and inadequate availability of patient information, unavailability of equipment, production pressure and hectic schedules, and physician fatigue. These errors are capable of leading a medical liability. On the other hand, the fear of penalties induces the use of procedures of defensive medicine, which are, by nature, unethical and increase the costs of medical practice. Besides, its use does not prevent future litigation.

Therefore, the authors conclude that the essential attitude to avoid medical liability is a good and ethical medical practice with the proper use of technology, based on knowledge of scientific evidence and ethical principles of medicine ‒ for the benefit of patients.

## Authors’ contributions

Ivan Dieb Miziara and Carmen Silvia Molleis Galego Miziara: These authors contributed equally to this work.

## Conflicts of interest

The authors declare no conflicts of interest.
